# CBP binding outside of promoters and enhancers in *Drosophila melanogaster*

**DOI:** 10.1186/s13072-015-0042-4

**Published:** 2015-11-24

**Authors:** Philge Philip, Ann Boija, Roshan Vaid, Allison M. Churcher, David J. Meyers, Philip A. Cole, Mattias Mannervik, Per Stenberg

**Affiliations:** Department of Molecular Biology, Umeå University, 901 87 Umeå, Sweden; Computational Life Science Cluster (CLiC), Umeå University, 901 87 Umeå, Sweden; Centre for Cellular and Molecular Biology, Uppal Road, Hyderabad, Telangana 500007 India; Department of Molecular Biosciences, The Wenner-Gren Institute, Stockholm University, 106 91 Stockholm, Sweden; Department Pharmacology and Molecular Sciences, The Johns Hopkins University School of Medicine, 725 North Wolfe Street, Baltimore, MD 21205 USA; Division of CBRN Security and Defence, FOI–Swedish Defence Research Agency, Umeå, Sweden

**Keywords:** CBP/p300, Drosophila melanogaster, Chromatin structure, Gene regulation, Insulators, Polycomb response elements

## Abstract

**Background:**

CREB-binding protein (CBP, also known as *nejire*) is a transcriptional co-activator that is conserved in metazoans. CBP plays an important role in embryonic development and cell differentiation and mutations in CBP can lead to various diseases in humans. In addition, CBP and the related p300 protein have successfully been used to predict enhancers in both humans and flies when they occur with monomethylation of histone H3 on lysine 4 (H3K4me1).

**Results:**

Here, we compare CBP chromatin immunoprecipitation sequencing data from *Drosophila* S2 cells with modENCODE data and show that CBP is bound at genomic sites with a wide range of functions. As expected, we find that CBP is bound at active promoters and enhancers. In addition, we find that the strongest CBP sites in the genome are found at Polycomb response elements embedded in histone H3 lysine 27 trimethylated (H3K27me3) chromatin, where they correlate with binding of the Pho repressive complex. Interestingly, we find that CBP also binds to most insulators in the genome. At a subset of these, CBP may regulate insulating activity, measured as the ability to prevent repressive H3K27 methylation from spreading into adjacent chromatin.

**Conclusions:**

We conclude that CBP could be involved in a much wider range of functions than has previously been appreciated, including Polycomb repression and insulator activity. In addition, we discuss the possibility that a common role for CBP at all functional elements may be to regulate interactions between distant chromosomal regions and speculate that CBP is controlling higher order chromatin organization.

**Electronic supplementary material:**

The online version of this article (doi:10.1186/s13072-015-0042-4) contains supplementary material, which is available to authorized users.

## Background

CREB-binding protein (CBP) is a transcriptional co-activator that is conserved in metazoans. In mammals, CBP shares functions with the paralogous adenovirus E1A binding protein p300. In *Drosophila*, there is only one CBP ortholog and it is called *nejire*, dCBP, CBP/p300, or CBP. CBP and p300 interact with multiple transcription factors and are thus associated with regulatory DNA sequences [1]. They also link enhancer-bound transcription factors and the basal transcription machinery. These proteins can affect the access of factors to DNA through their histone acetyltransferase activity [2]. Loss of CBP/p300 gene function leads to cell death in flies, mice and worms [3]. CBP plays an important role in embryonic development and cell differentiation [3] and is associated with some diseases. For example, heterozygosity of p300 or CBP in humans causes Rubinstein–Taybi syndrome which is characterized by broad thumbs and distinctive facial features [4]. CBP acts as a tumor suppressor in mouse where its inactivation leads to tumor formation. CBP and p300 are disrupted by chromosomal translocations with mixed lineage leukemia (MLL) or other partners in some leukaemias and are targets of DNA tumor virus transforming proteins [5]. Recent exome sequencing has revealed frequent inactivating mutations in CBP and p300 in B cell lymphoma, relapsed acute lymphoblastic leukemia, bladder carcinoma, and small-cell lung cancer [6].

Today, over 400 interaction partners have been described for CBP including proteins from all major transcription factor families. Many CBP binding sites are localized in sites known as HOT sites which are regions in the genome that bind multiple transcription factors [7]. CBP bound regions are generally DNase I hypersensitive and most are found in promoters, introns and intergenic regions [8]. Studies involving *Drosophila* embryos suggest that CBP is targeted preferentially by some transcription factors in the genome [7]. In early embryos, CBP co-occupies genomic locations with the Rel-family transcription factor dorsal [7]. Early studies on p300 in mammalian cells show that p300 has an affinity for specific DNA sequences that are recognized by the Rel protein nuclear factor kappa B (NF-_K_B) [9]. This suggests that the association of CBP/p300 with Rel-family proteins is evolutionarily conserved. At active genes, CBP can acetylate several lysines on the histones, predominantly histone H3 on lysine 27 (H3K27ac), histone H3 on lysine 18 (H3K18ac), and histone H4 on lysine 8 (H4K8ac) [10, 11]. It can also acetylate transcription factors that recruit RNA polymerase II, function as a scaffold for recruiting other proteins and help establish a pre-initiation complex by interacting with transcription factor IIB and hypophosphorylated RNA polymerase II [12]. Other histone marks, such as acetylation of histone H3 on lysine 23 (H3K23ac) and 56 (H3K56ac), are also influenced by CBP [13, 14]; the presence of H3K23ac is associated with ecdysone induced gene activation [13] and H3K56ac has a critical role in the packaging of DNA into chromatin following DNA replication and repair [14]. Additionally, CBP regulates DNA replication in *Drosophila* ovarian follicle cells and Kc cells [15, 16].

In humans, CBP/p300 binding regions that are outside of genes often overlap with H3K4me1 and are a signature of transcriptional enhancers [17]. The genomic occupancy of CBP, which has been detected by ChIP-seq experiments, has been used to predict novel enhancers both in human and flies [18, 19]. CBP bound regions from different tissues can be used to identify enhancers that are active in a tissue specific manner [19]. The H3K27ac mark distinguishes active from poised enhancers [20–22]. Since CBP is responsible for H3K27 acetylation, it has been presumed that poised or inactive enhancers lack CBP. However, CBP binds to many silent regions without histone acetylation [21, 22]. CBP binding that does not result in histone acetylation occurs at some silent genomic regions lacking active transcription [7], and CBP occupancy can in fact be detected at poised or inactive enhancers containing H3K27me3 [21, 22].

Although CBP occupancy generally correlates with active genes, silent genomic regions with Polycomb-mediated H3K27me3 prevent histone acetylation but do not inhibit CBP binding [7]. Although many protein complexes are involved, Polycomb repression is mainly mediated by the canonical Polycomb repressive complexes 1 and 2 (PRC1 and PRC2) together with the DNA binding pleiohomeotic (Pho) repressive complex [23]. Studies on the antagonistic switch between H3K27ac and the H3K27me3 mark show that CBP is involved in the switch between the repressed and active chromatin states [10, 24]. CBP also interacts with the Trithorax (Trx) group of chromatin modifiers to maintain the active state of Polycomb target genes [10].

Though most CBP binding sites in the genome are at promoters and enhances, not all genomic positions bound by CBP are promoters or enhancers. In this paper, we have investigated the full range of CBP bound regions to determine if CBP is involved in any additional processes that have yet to be identified. Our main objective was to classify the local chromatin environments where CBP is found. We also wanted to identify both the proteins and histone modifications that potentially interact with CBP and may modulate CBP activity and recruitment to chromatin. We found that CBP binding sites can be classified into Polycomb repressed regions, inactive enhancers, active enhancers, active promoters and insulators. At some insulators, we discovered that CBP functions to prevent repressive H3K27 methylation from spreading into active genes. Our results also suggest that CBP has a role in chromatin opening, DNA replication and chromosomal interactions.

## Results and discussion

### Classification of CBP bound chromatin identifies active promoters and enhancers

**Fig. 1:**
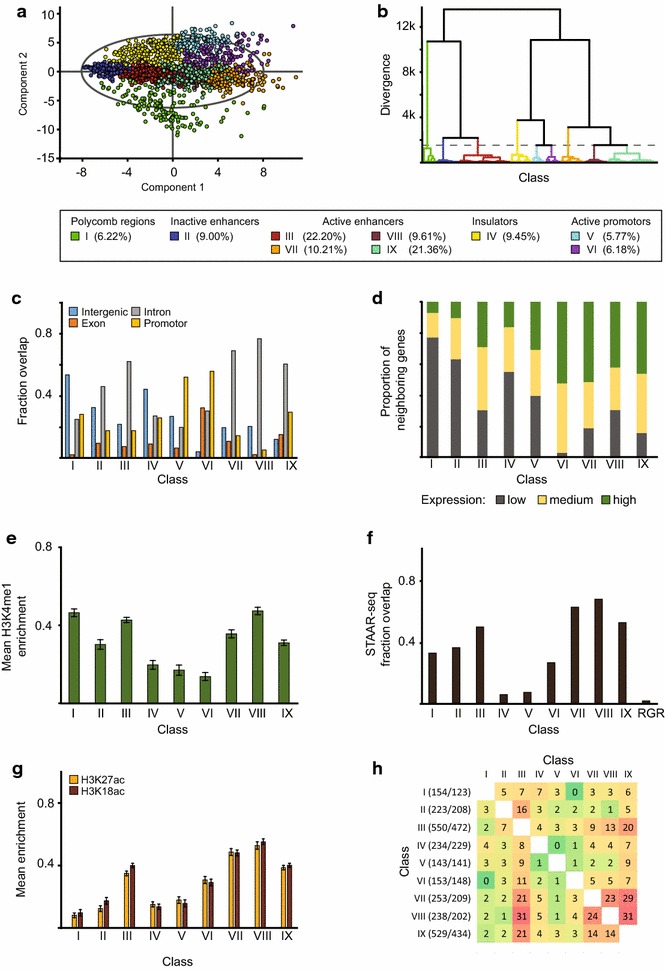
Classification of CBP binding sites and identification of promoter and enhancer classes. **a** Score plot of the first two components from principal component analysis (PCA) of 42 protein binding values within CBP binding sites. **b** Hierarchical clustering of CBP binding sites based on the four significant principal components. The* dashed line* indicates the cut-off used to define nine classes. These classes represent regions with a distinct combination of factors bound, and correspond to various kinds of* cis*-regulatory regions. The percentages of CBP binding regions from each class are given in brackets. **c** Fraction of CBP peaks in the nine classes overlapping different gene features. **d** Fraction of CBP peaks in the nine classes associated with genes divided into three levels of expression. **e** H3K4me1 enrichment within 500 bp around the CBP peak centres in the nine CBP classes. **f** Fraction of CBP regions in the nine classes and in random genomic regions (RGR) that are associated with STARR-seq enhancer peaks. **g** H3K27ac and H3K18ac enrichment within 500 bp around CBP peak centres in nine classes of CBP. Values were scaled so that a value of zero corresponds to the genomic mean and a value of one to the genomic maximum in** e** and** g**.* Error bars* represent 95 % confidence intervals. **h** Percentage overlap between the genes associated with each class. Each row represents the percentage of the genes associated with that class that is also associated with each of the other eight classes. Within parenthesises the number of CBP regions and number of unique genes assigned to each class

Using our previously characterized C-terminal CBP antibody [7], we mapped CBP by ChIP-seq across the *Drosophila melanogaster* S2 cell-line genome. Based on these data, 2477 high-confidence binding sites of CBP were defined. We also used proteins and histone modifications mapped by modENCODE [25] in these cells (42 proteins and 27 histone modifications). For all 42 proteins, we calculated the amount of binding at CBP sites (within 150 bp) (see “Methods” section). Using the protein binding data (CBP binding regions as observations and the levels of enrichment of the 42 proteins at the CBP regions as variables), we classified all CBP binding sites into nine distinct classes using principal component analysis (PCA) followed by hierarchical clustering (HCA) (Fig. 1a, b). Although the PCA classification yielded fairly distinct classes, binding of several of the 42 proteins are shared between some classes. The different classes should rather be interpreted as sub-sets of regions with a distinct combination of factors bound.

To find out where the CBP regions of the nine classes are located in the genome, we mapped their positions in relation to the following gene features: promoters, exons, introns and intergenic regions. Classes V and VI CBP binding sites map to promoters of genes that tend to be highly expressed (Fig. 1c, d). We therefore conclude that Classes V and VI are active promoters. Class VI binding sites have more RNA polymerase II (Pol II) compared to Class V sites and are associated with higher expression levels (Additional file 1: Figure S1; Fig. 1d). In addition, factors such as suppressor of variegation 3–7 (Su(var)3–7), Bre1, CCCTC-binding factor (CTCF) and centrosomal protein 190 kD (CP190) were found to bind more strongly to Class V than to Class VI regions (Additional file 1: Figure S1).

Since CBP is commonly used as a marker for enhancers, we compared the CBP binding classes to H3K4me1 regions and to enhancers that were experimentally identified by self-transcribing active regulatory region sequencing (STARR-seq) in S2 cells [25, 26]. We found that Classes III, VII, VIII and IX overlap with STARR-seq enhancers, have H3K4me1 and are mostly located in introns (Fig. 1c, e, f). They are also situated in highly active genes (Fig. 1d). Based on these observations we conclude that Classes III, VII, VIII and IX represent active enhancers.

We next looked at the differences between these four active enhancer classes and found that Classes VII, VIII and IX were bound by different combinations of factors (Additional file 1: Figure S1), such as the transcription factor GAGA factor (GAF), subunits of the Nucleosome Remodeling Factor complex (NURF301), the chromatin complex FACT (Spt16), the Nucleosome Remodeling Deacetylase complex (dMi-2) and histone deacetylase 1 (RPD3). Interestingly, the GO annotations for genes closest to the CBP sites differed for each of these classes (Additional file 2: Table S1). For example, Class VII is associated with genes involved in “positive regulation of transcription” and Class VIII is enriched with genes involved in “negative regulation of transcription”. Class IX associated genes are mainly involved in metamorphosis while Class III is not associated with any significant GO enrichment and contains lower levels of all of the modENCODE factors studied. Although it is hard to speculate on the function of Class III enhancers, they clearly have a very different chromatin composition compared to the other enhancer classes.

We note that Classes I and II also show enhancer-like characteristics (Fig. 1e, f), although Classes I and II are mainly situated close to silent genes. These regions show low levels of H3K18ac and H3K27ac, indicating that they may be inactive enhancers (Fig. 1g). Interestingly, we find that active enhancers (Classes III, VII, VIII, IX) have higher levels of H3K18ac and H3K27ac than active promoters (Classes V and VI) (Fig. 1g).

Since several enhancers could potentially be associated with the same gene, we calculated the percentage overlap between the genes in each class (Fig. 1h). 9–31 % of the genes in Classes III, VII, VIII and IX overlap, indicating that despite differences in GO enrichment between the classes, there are many examples where several different types of enhancers map to the same gene. This is to be expected as it has been estimated that there are about four enhancers per expressed protein-coding gene that are active during embryogenesis in *Drosophila* [27].

### CBP occupies Polycomb response elements

**Fig. 2:**
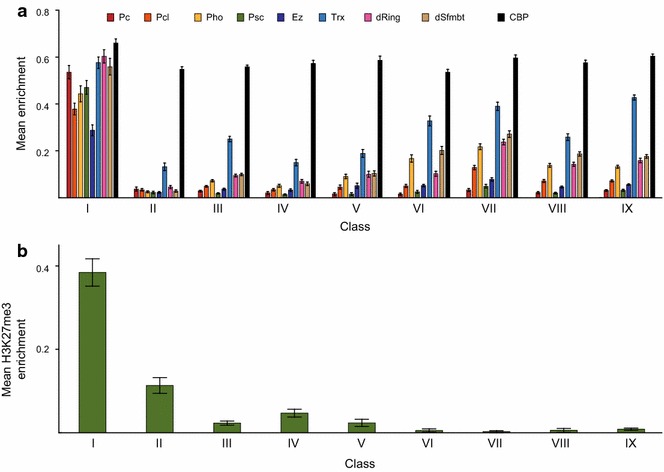
Enrichment of Polycomb factors and H3K27me3 in class I CBP regions. **a** Polycomb and Trx complex factors as well as CBP enrichment in 300 bp around CBP peak centres in the nine classes of CBP regions. **b** H3K27me3 enrichment within 500 bp around CBP peak centres in the nine classes of CBP regions. Values were scaled so that a value of zero corresponds to the genomic mean and a value of one to the genomic maximum. *Error bars* represent 95 % confidence intervals

Our search strategy revealed that, as expected, CBP binds to active promoters (Classes V and VI) and active enhancers (Classes III, VII, VIII and IX). We were also interested in exploring the features of the other classes. Surprisingly, we find that Class I is highly enriched in Polycomb factors such as Polycomb (Pc), Enhancer of zeste (Ez), Pho, Scm-related gene containing four mbt domains (dSfmbt), Sex combs extra (dRing), Polycomb-like (Pcl), Posterior sex combs (Psc), and Trx (Fig. 2a; Additional file 1: Figure S1). In fact, 66 % of Class I sites overlap with Polycomb Response Elements (PREs) in the repressed state (defined in [28] in the S2 related cell-line SG4). Out of the 200 defined PREs in Schwartz et al. [28], 94 % have at least twofold enrichment of CBP. As expected, Class I sites have high H3K27me3 as well as low acetylation levels (Figs. 1g and 2b). The genes close to Class I sites are expressed weakly or not all (Fig. 1d). Although it has previously been shown that CBP can bind to both active enhancers and to inactive enhancers containing H3K27me3 [7, 21, 22] such regions are often embedded within H3K27me3 domains (usually up to several hundreds of kbp long [28]). Importantly, we demonstrate here that CBP occupies the short PRE elements (a few hundred bp in length) that initially recruit the Polycomb repressive complexes, and that CBP occupancy of PREs does not displace Polycomb complexes or lead to gene activation. At these sites, CBP may not antagonize Polycomb repression and H3K27me3 as it does on a global level [10, 24, 29].

We next compared the enrichment levels of all proteins to those of CBP (±150 bp from the CBP peak centre). Within Class I binding sites, the enrichment levels of CBP correlate (Spearman correlation, *p* < 0.05) with the enrichment levels of Pho and dSfmbt (members of the Pho repressive complex) but not with any PRC1 or PRC2 components, indicating that CBP might interact with the Pho repressive complex. Of all of the classes, the strongest CBP sites in the genome are found in Class I (Fig. 2a). Interestingly, Class I regions have a high overlap with STARR-seq enhancers and have high H3K4me1 levels (Fig. 1e, f). CBP has been implicated in Polycomb/trithorax regulation. CBP interacts with Trx [30] and was thought to be involved in maintaining the active state of Pc/Trx regulated genes because it is a histone acetyl transferase [10]. Here, we show that CBP occupies PREs even when they are in the repressed state which provides evidence that CBP can remain attached to chromatin without active histone acetylation. It remains to be investigated, however, if CBP acetylates non-histone targets at PREs or if the histone acetyltransferase (HAT) activity is blocked.

### Class 2 CBP sites are devoid of known proteins and gene activity

**Fig. 3:**
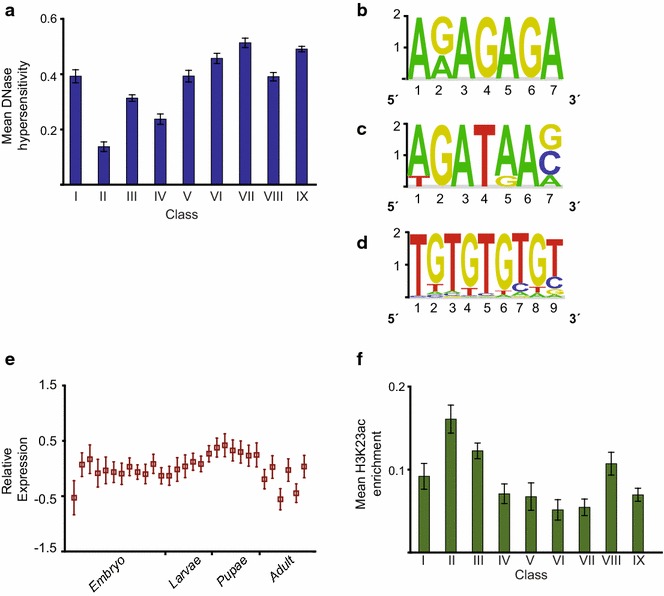
Class II CBP regions have low DNase hypersensitivity and become active late in development. **a** DNase hypersensitivity within 150 bp of the CBP peak in the nine classes of CBP regions. **b** GAGA, **c** GATAe and **d** GTGT motifs identified from CBP Class II regions. **e** Developmental expression of genes proximal to Class II CBP peaks. For each gene the relative change in expression from the previous time point was used. Values were linearly adjusted so that the global mean was zero. The embryo time points range from 0–24 h (in 2 h intervals) and the larval stages are as follows: larva L1, L2, L3 (12 h), L3 (puff stage 1–2), L3 (puff stage 3–6), L3 (puff stage 7–9). The pupae stages are: white pupae new, 12 h, 24 h, and 2, 3 and 4 days post white pupae. The adult time points are as follows: males 1 day, males at 5 days, males at 30 days, females at 1 day, females at 5 days and females at 30 days. **f** H3K23ac enrichment within 500 bp around CBP peak centres in the nine classes of CBP regions. Values were scaled so that a value of zero corresponds to the genomic mean and a value of one to the genomic maximum in **a** and **f**. *Error bars* represent 95 % confidence intervals

Class II CBP sites generally lacked mapped proteins and histone modifications from modENCODE (Additional file 1: Figure S1). Class II regions also are less DNAse hypersensitive compared to the other classes (Fig. 3a). This class has some overlap with STARR-seq enhancers and has regions that are slightly enriched in H3K4me1 (Fig. 1e, f). Since we found no enrichment of any mapped proteins, we performed sequence motif analysis of Class II regions using word counting and multivariate modeling as described in [31]. Sequence analysis revealed that Class II regions were rich in GAGA motifs which suggests that they have the potential to bind GAF. We also found that the GATAe and the GTGT motifs were enriched in Class II regions (Fig. 3b–d) which is intriguing because similar dinucleotide repeat motifs are important for enhancer function in *D. melanogaster* [32].

The genes close to Class II CBP sites become most active during pupal stages of development (Fig. 3e), and are enriched for the GO terms “plasma membrane” and “post-embryonic development” (Additional file 2: Table S1). We speculate that Class II CBP sites represent poised enhancers that are not yet active and note that CBP is recruited to these regions earlier than other activating factors. CBP HAT activity appears to be blocked from acetylating H3K18 and H3K27 when these enhancers are inactive (Fig. 1g). To investigate if these enhancers become active later in development, we calculated H3K27ac enrichment at Class II sites across the developmental time points where modENCODE has mapped this modification by ChIP-seq (Additional file 3: Figure S2). However, the H3K27 acetylation levels at these sites are very low at all time points tested. This could be because the relevant time point has not been mapped, or that these enhancers are active only in very few cells and thus the signal becomes undetectable in the mixture of cells assayed. Alternatively, the Class II regions do not represent “canonical” enhancers. With the available data we cannot distinguish between these alternatives. Interestingly, although the H3K23ac data we used are noisy, we observed that the H3K23ac modification is highest in Class II (Fig. 3f). Perhaps the MYST-family HAT Enok (KAT6) that acetylates H3K23 is enriched at these sites [11, 33], or the inability to acetylate H3K18 and H3K27 directs CBP activity to H3K23 instead. Indeed, CBP is the most promiscuous of the HATs, and reduced global acetylation of one histone residue results in compensatory acetylation of other lysine residues [11]. For example, knock-down of KAT6 reduces H3K23ac but increases H3K18ac, pointing to an interplay between KAT6 and CBP [11]. Proteins that have yet to be mapped may redirect CBP HAT activity to H3K23 or recruit KAT6 to these regions.

### CBP binds to insulators and regulates their activity

Class IV CBP sites are highly enriched in several insulator factors (Additional file 1: Figure S1). Several different types of insulators were recently characterized in *Drosophila* by Schwartz et al. [34]. When we compare the Class IV regions with the insulator classes defined by [34], about 50 % overlap with CP190 and suppressor of Hairy wing (Su-Hw) type insulators. When studying all genomic sites of some of the major insulator classes defined in [34], we observe that CBP is enriched at least twofold over background at most of these sites (Additional file 4: Figure S3). This implies that some CBP binding is present at most insulators in the genome. Note that we use a stringent cut-off to define CBP binding sites and therefore most of the insulators will not be included in the classification in Fig. 1.

**Fig. 4:**
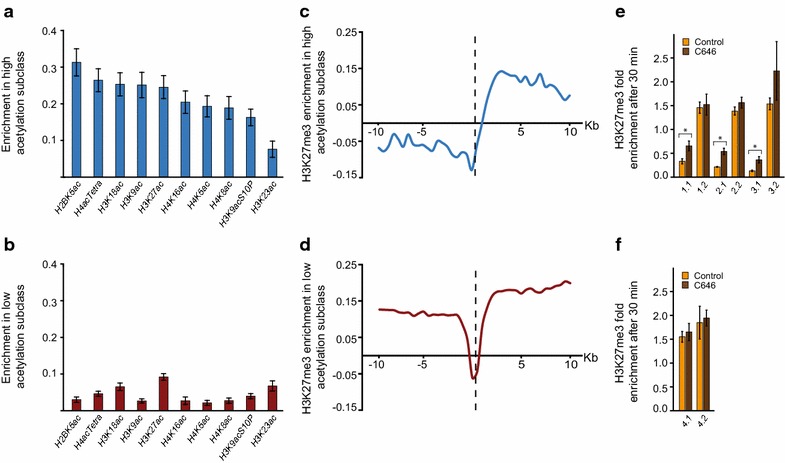
Class IV CBP regions with histone acetylation represent functional insulators. **a** Enrichment of different histone acetylations within 500 bp around the CBP peak centres in Class IV. The Class IV regions were divided into two sub-classes, **a** and **b**, based on a PCA using all histone modifications (*n* = 27). *Error bars* represent 95 % confidence intervals. **c**, **d** Average enrichment of the H3K27me3 mark within 10 kb around the CBP peak centres in the two sub-classes of Class IV CBP regions (sub-class with high levels c and low levels d of acetylation). Only Class IV regions within 10 kb of an H3K27me3 domain were considered. The regions were oriented so that the highest level of H3K27me3 was to the right of the CBP peak centre (*vertical dashed line*) in the plots. Values were scaled so that a value of zero corresponds to the genomic mean and a value of one to the genomic maximum. **e**, **f** ChIP-qPCR (3–4 biological replicates) using H3K27me3 antibodies (normalized to H3) after 30 min of CBP HAT inhibition (C646, in *brown*) and control treatment (C37, in *orange*) at regions surrounding Class IV sites with high acetylation (**e**, regions 1–3) and low acetylation (**f**, region 4). Regions are oriented so that the left pair of primers (x.1) are located at the side with lower acetylation [as in (**c**, **d**)]. *Error bars* represent standard error of the mean

When further analysing the Class IV sites we observed that some sites have high while others have low levels of histone acetylation. We, therefore, performed a PCA using Class IV sites as observations and all histone modifications as variables. Interestingly, this analysis resulted in two main subclasses one of which had lower acetylation levels with the exception of H3K23ac (Fig. 4a, b). To determine if these insulator-like regions have insulator activity, we selected all intergenic Class IV regions (most Class IV regions are intergenic) overlapping H3K27me3 regions in the genome (*n* = 184) and plotted the average methylation 10 kb up and downstream. Regions were oriented so that the highest level of H3K27me3 was to the right of the Class IV site. Interestingly, the Class IV CBP sites with high levels of acetylation appear to block the spread of H3K27me3, whereas Class IV sites with low levels of acetylation do not appear to have this capacity (Fig. 4c, d).

To investigate whether the HAT activity of CBP is directly involved in the regulation of insulator activity at these sites, we treated S2 cells with the CBP inhibitor C646 [35] or with C37 (a compound similar to C646 that shows no effect on CBP HAT activity, [35]) and used H3K27me3 antibodies in ChIP-qPCR. We selected three Class IV regions with high acetylation levels that also showed high H3K27me3 enrichment proximal to the insulator, as well as one control region (a Class IV region with low acetylation levels but with high proximal H3K27me3 enrichment). After 30 min of C646 treatment, the H3K27me3 levels significantly increased (2.4-fold on average) on the side of the insulators that had lower initial H3K27me3 levels at all three Class IV regions with high acetylation (*T* test, *p* < 0.05, Fig. 4e). This shows that the H3K27me3 modification is now able to spread past the insulator. No change in H3K27me3 was observed at the Class IV region with low acetylation (Fig. 4e) which is consistent with the genome-wide data where no blocking of H3K27me3 was seen in this subclass. Given the relative short treatment (30 min), we suggest that CBP is likely to directly regulate insulator activity, at least at the three regions studied here.

We note that Class V, and to some degree Class VI, are enriched in insulator proteins (Additional file 1: Figure S1). These classes are active promoters, and it has been shown that some paused promoters have insulator activity [36]. We speculate that these promoters need insulation from neighboring repressors or repressive chromatin. Indeed, Class V promoters are, on average, closer to H3K27me3 domains than other active promoters bound by CBP (~20 and ~35 kb, respectively, *p* < 0.05). We therefore propose that CBP plays an important role in insulator function and suggest that the HAT activity of CBP regulates insulating activity in some instances.

### CBP is found at genomic regions involved in long range interactions

**Fig. 5:**
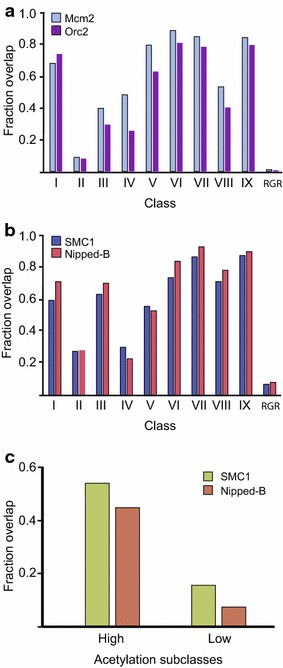
CBP bound regions strongly overlap replication factors and cohesin components. **a** The fraction of CBP regions from the nine classes and from random genomic regions (RGR) that overlap with replication factors Orc2 and Mcm2. **b** The fraction of CBP regions that overlap bound regions of the cohesin complex components SMC1 and Nipped-B. **c** Fraction of CBP regions in the two sub-classes of Class IV overlapping cohesin components SMC1 and Nipped-B

Once the distinctive features of each CBP binding class had been identified, we then examined common features of CBP bound regions across the genome. By analysing the mean enrichment of all 42 modENCODE factors in all classes, we observed that the DNA replication factors origin recognition complex subunit 2 (Orc2) and minichromosome maintenance 2 (Mcm2) were found in all classes except Class II. This was confirmed by overlapping CBP regions with defined regions of Orc2 and Mcm2 binding (Fig. 5a). CBP is known to co-localize with origins of replication and may contribute to their regulation [15].

Taken together, our results show that CBP is bound at genomic sites with a wide range of functions. A common feature of these functional elements is that they physically interact with other chromosomal regions. For example, promoters, enhancers and insulators interact via loop formations that are functionally important [37, 38], while PREs interact with the promoters of genes that are to be repressed [39]. Furthermore, the origins of replication appear to form “replication factories” in eukaryotes [40]. The cohesin complex was recently shown to be important for chromosomal interactions and in the formation of chromosomal domains [41]. We, therefore, examined the overlap between the CBP classes and the cohesin components structural maintenance of chromosomes 1 (SMC1) and Nipped-B, which have been mapped in the SG4 cell-line that is closely related S2 cells (data from [42]). All classes except Class II and Class IV show a strong overlap with the defined cohesin binding sites (Fig. 5b). Since Class II regions represent inactive enhancers they are not expected to interact with their target promoters. Class IV regions, however, are insulators that we expect to be involved in chromosomal interactions. Indeed, when we looked at the high acetylation subclass of Class IV insulators, we found that they overlap with cohesin sites (Fig. 5c). In contrast, Class IV sites that lack histone acetylation and that fail to prevent spreading of H3K27me3 show little overlap with cohesin (Fig. 5c).

## Conclusions

Our study shows that CBP is found at active promoters and enhancers as previously demonstrated [8, 17]. In addition, we show that CBP is recruited to inactive regions with enhancer-like features that are associated with genes that become active at other developmental stages. We also show that the strongest CBP sites in the genome are found at silent PREs where they correlate with the binding of the Pho repressive complex. Since CBP is also found at promoters of Polycomb regulated genes that are active [10], it will be interesting to examine further the involvement of CBP in Polycomb regulation. Another novel finding is that CBP binds to many, if not most, insulators across the genome and regulates insulating activity through its HAT activity at a subset of these insulators. We speculate that CBP may control higher order chromatin organization at all types of functional genomic sites, and that this could be part of the explanation for its cell-autonomous lethal phenotype.

## Methods

### PCA analysis

To conduct PCA analysis, a matrix was generated with CBP bound regions as observations and the amount of binding of different proteins in CBP bound regions as variables. To calculate the amount of binding of different proteins at CBP sites, the top three consecutive binding values for each protein within 150 bp of the CBP peak centre was averaged. The genomic mean was used if three binding values were not available. All computations were done in log2 scale. PCA was applied to the data after unit variance scaling. Hierarchical clustering was then applied on the first four significant components of PCA, to define clusters of CBP bound regions. Ward clustering was used to calculate tree distances. Nine classes of CBP bound regions were defined in the hierarchical cluster tree.

### Expression analysis and annotation

Flybase annotation Release 5.32 [43] was used for all annotation. S2 cell-line expression values from [44] was grouped into three equally sized bins (low expressed/unexpressed, medium expressed and high expressed). The closest transcription start site was used to assign a gene to each CBP peak for the expression analysis. Gene ontology analysis was done using DAVID [45, 46].

### Comparing CBP to other data

For each dataset except for the histone modifications, the average of the highest three consecutive values within 150 bp of the CBP peak center was used. For the histone modifications, 500 bp from the CBP peak center was considered. If the calculated value was below the genomic mean for the dataset, then the genomic mean was used instead. All modENCODE data (42 proteins listed in Additional file 1: Figure S1, 27 histone modifications, DNAse hypersensitivity and expression data produced in *Drosophila* S2 cells as well as H3K27ac and expression in different developmental stages) was obtained from modMine (http://intermine.modencode.org/). Two additional proteins, Trx and M1BP (GEO entries GSM604729, GSM1208162), that were not used in the classification, were included in the heatmap (Additional file 1: Figure S1) for comparison. The Cluster tool [47] was used to generate the heatmap.

### DNA motif analysis

Sequences of Class II CBP bound intronic regions and random control genomic intron sequences were submitted to multivariate DNA motif analysis as described in [31]. Control sequences with an A/T content falling outside the range of the A/T contents of the CBP bound regions was excluded.

### ChIP

*Drosophila* S2 cells were grown to a density of 0.2–1 × 10^7^ cells/ml and fixed in 1 % formaldehyde for 15 min at ambient temperature. The reaction was quenched by 0.16 M glycine pH 7.0 for 5 min and washed in PBS. Cells were sequentially washed with ChIP A (10 mM Hepes pH 7.6, 10 mM EDTA pH 8.0, 0.5 mM EGTA pH 8.0, 0.25 % Triton X100) and ChIP B (10 mM Hepes pH 7.6, 100 mM NaCl, 1 mM EDTA pH 8.0, 0.5 mM EGTA pH 8.0, 0.01 % Triton X100) for 10 min at 4 °C followed by resuspension in Sonication buffer (50 mM Hepes, 140 mM NaCl, 1 mM EDTA, 1 % Triton, 0.1 % sodium deoxycholate, 0.1 % SDS, supplemented with proteinase inhibitor tablets, Roche) to a final concentration of 5–10 × 10^7^ cells/ml. Nuclei were sonicated for 15 min using a Bioruptor (Diagenode), rotated for 10 min followed by centrifugation for 10 min at 13,000 rpm at 4 °C.

A mix of Protein A and G Dynabeads (Invitrogen) blocked with only BSA (Sigma Aldrich) for ChIP-sequencing or BSA (1 mg/ml) and salmon sperm DNA (1 mg/ml) for ChIP-qPCR were mixed with indicated antibodies. Beads and antibodies were incubated for at least 2 h followed by the addition of 0.5–1 × 10^7^ cells.

Chromatin and antibody bead complexes were formed during at least 2 h followed by 5 min washes with sonication buffer (50 mM Hepes, 140 mM NaCl, 1 mM EDTA, 1 % Triton, 0.1 % sodium deoxycholate, 0.1 % SDS), WashA (as sonication buffer, but with 500 mM NaCl), WashB (20 mM Tris pH 8, 1 mM EDTA, 250 mM LiCl, 0.5 % NP-40, 0.5 % sodium deoxycholate) and TE.

Beads were resuspended in Elution buffer (50 mM Tris pH 8, 50 mM NaCl, 2 mM EDTA, 0.75 % SDS, 20 µg/ml RNase A, 20 µg/ml glycogen) in a new tube. Cross-linking was reversed at 68 °C for at least 4 h and proteins removed by Proteinase K. DNA was purified with phenol–chloroform, ethanol precipitated and finally resuspended in 200 µl 0.1 × TE.

Chromatin immunoprecipitation samples were analyzed by qPCR or sequenced at the Uppsala Genome Center. 2 µl of DNA was used as template for qPCR, which was run in duplicates using 300 nM primers and EvaGreen (Solis BioDyne) on a CFX96 Real-Time system (BioRad). Average Cq was calculated for each ChIP sample and compared to input. To account for the background of each individual ChIP, normalization was made to two intergenic sites devoid of known histone modifications and chromatin factors. ChIP values were further normalized to the total amount of histone H3.

Ten ChIP samples were pooled and used for SOLiD (TM) ChIP-Seq Library preparation, size selection (100–150 bp + adapters 90 bp) and sequenced using SOLiD4 50 bp fragment run.

### ChIP-seq data processing

CBP ChIP-seq reads were mapped to the *D. melanogaster* genome (release 5) and only those with unique map sites were retained. A log2-ratio was calculated between the IP and the input samples read densities. After median smoothing with 100 bp windows, high-confidence binding sites were identified as described in [7]. In brief, regions of at least 200 bp with a log2-enrichment of more than the 95th percentile were considered high-confidence binding sites. The CBP data is available at Gene Expression Omnibus (GEO, Acc. No. GSE64464).

### Antibodies

Two affinity purified antibodies raised against CBP, one in guinea-pig [48, 49] and one in rabbit [7] was used for ChIP-sequencing. The following antibodies were used in ChIP-qPCR, H3K27me3 (Abcam, ab6002) and H3 (Abcam, ab1791).

### Drug treatment of S2 cells

2 × 10^6^ cells/ml of S2 cells were centrifuged and dissolved in FCS free media. 30 µM of CBP inhibitor (C646) or control drug (CM37) in DMSO was added to the cells for 30 min before ChIP.

## Additional files

10.1186/s13072-015-0042-4 Enrichment of chromatin factors in the nine classes of CBP regions. Values were scaled so that a value of zero (black) corresponds to the genomic mean and a value of one (red) to the genomic maximum. Values above the class numbers represent the number of CBP regions in each class.
10.1186/s13072-015-0042-4 GO analysis of genes close to the nine classes of CBP regions. Enrichment of gene ontology clusters for each of the nine classes.
10.1186/s13072-015-0042-4 H3K27ac at Class II sites in different developmental stages. Values were scaled so that a value of zero corresponds to the genomic mean and a value of one to the genomic maximum. Error bars represent 95 % confidence intervals.
10.1186/s13072-015-0042-4 IP versus input enrichment of CBP in four classes of insulator regions. Regions are sorted by CBP enrichment.

## Abbreviations

CBP: CREB-binding protein
ChIP-seq: chromatin immunoprecipitation sequencing
CP190: centrosomal protein 190kD
dRing: sex combs extra
dSfmbt: scm-related gene containing four mbt domains
Ez: enhancer of zeste
GAF: transcription factor GAGA
H3K4me1: monomethylated histone H3 lysine 4
H3K18ac: acetylated histone H3 lysine 18
H3K23ac: acetylated histone H3 lysine 23
H3K27ac: acetylated histone H3 lysine 27
H3K27me3: trimethylated histone H3 lysine 27
H3K56ac: acetylated histone H3 lysine 56
HAT: histone acetyltransferase
HCA: hierarchical clustering
Mcm2: minichromosome maintenance 2
MLL: mixed lineage leukemia
NF-_k_B: nuclear factor kappa B
NURF301: enhancer of bithorax
Orc2: origin recognition complex subunit 2
Pc: polycomb
PCA: principal component analysis
PCL: polycomb-like
Pho: pleiohomeotic
PRE: polycomb response element
Psc: posterior sex combs
RPD3: histone deacetylase 1
SMC1: structural maintenance of chromosomes 1
STARR-seq: self-transcribing active regulatory region sequencing
Su-Hw: suppressor of Hairy wing
Su(var)3–7: suppressor of variegation 3–7
Trx: trithorax
