# Erratum to: CBP binding outside of promoters and enhancers in *Drosophila melanogaster*

**DOI:** 10.1186/s13072-016-0088-y

**Published:** 2016-09-12

**Authors:** Philge Philip, Ann Boija, Roshan Vaid, Allison M. Churcher, David J. Meyers, Philip A. Cole, Mattias Mannervik, Per Stenberg

**Affiliations:** 1 Department of Molecular Biology, Umea Umeå University, 901 87 Umeå, Sweden; 2 Computational Life Science Cluster (CLiC), Umeå University, 901 87 Umeå, Sweden; 3 Centre for Cellular and Molecular Biology, Uppal Road, Hyderabad, Telangana 500007 India; 4 Department of Molecular Biosciences, The Wenner-Gren Institute, Stockholm University, 106 91 Stockholm, Sweden; 5 Department Pharmacology and Molecular Sciences, The Johns Hopkins University School of Medicine, 725 North Wolfe Street, Baltimore, MD 21205 USA; 6 Division of CBRN Security and Defence, FOI–Swedish Defence Research Agency, Umeå, Sweden

## Erratum to: Epigenetics & Chromatin (2015) 8:48 DOI 10.1186/s13072-015-0042-4

After the publication of this work [1], it was noticed that the funding source was inadvertently omitted from the acknowledgements. Please see the corrected acknowledgements statement.
