# Supplementary figures and images for: CBP binding outside of promoters and enhancers in Drosophila melanogaster
Source: Epigenetics Chromatin. 2015 Nov 24;8:48. doi: 10.1186/s13072-015-0042-4 (PMC4657240; doi:10.1186/s13072-015-0042-4)

Supplementary Figure 1

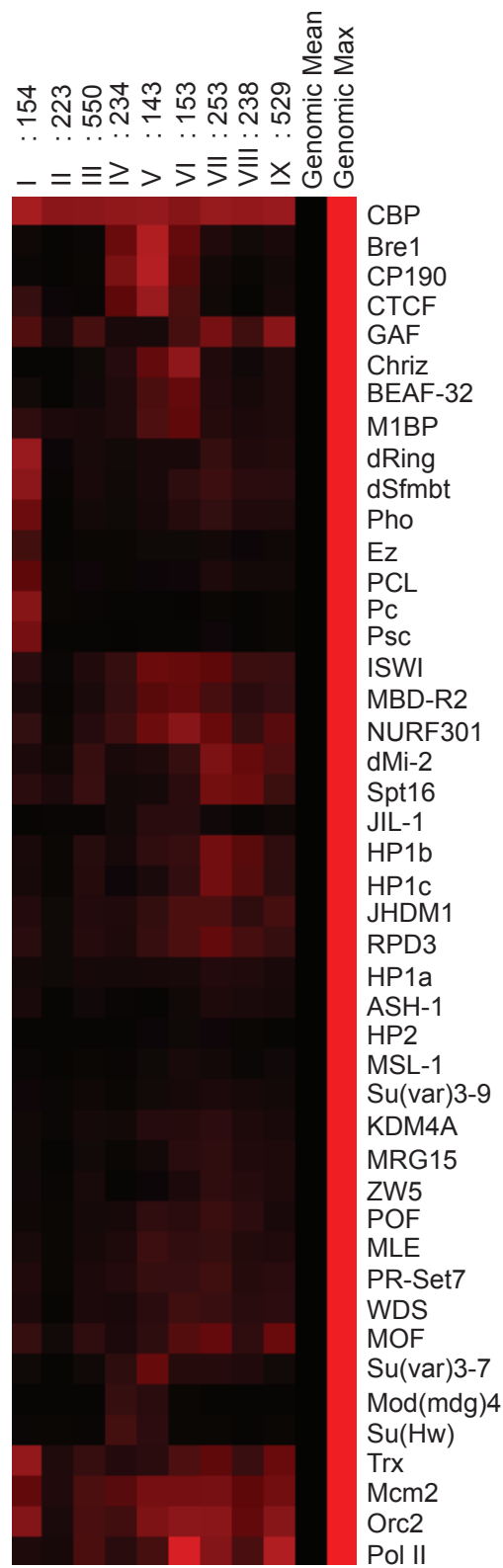

Supplement: Supplementary file 1 — 10.1186/s13072-015-0042-4 Enrichment of chromatin factors in the nine classes of CBP regions. Values were scaled so that a value of zero (black) corresponds to the genomic mean and a value of one (red) to the genomic maximum. Values above the class numbers represent the number of CBP regions in each class. [file 13072_2015_42_MOESM1_ESM.pdf]

Supplementary Figure 2

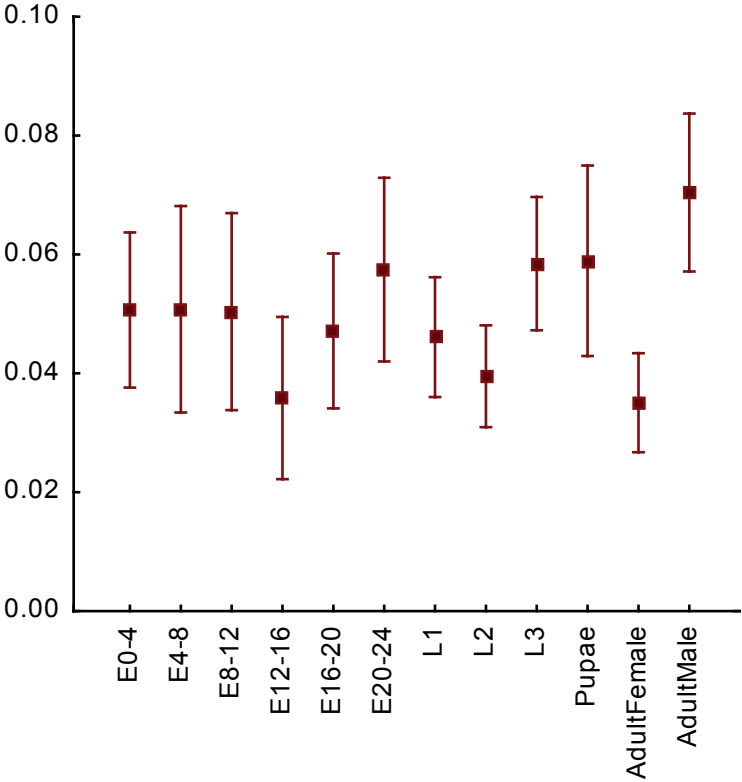

Supplement: Supplementary file 3 — 10.1186/s13072-015-0042-4 H3K27ac at Class II sites in different developmental stages. Values were scaled so that a value of zero corresponds to the genomic mean and a value of one to the genomic maximum. Error bars represent 95 % confidence intervals. [file 13072_2015_42_MOESM3_ESM.pdf]

Supplementary Figure 3

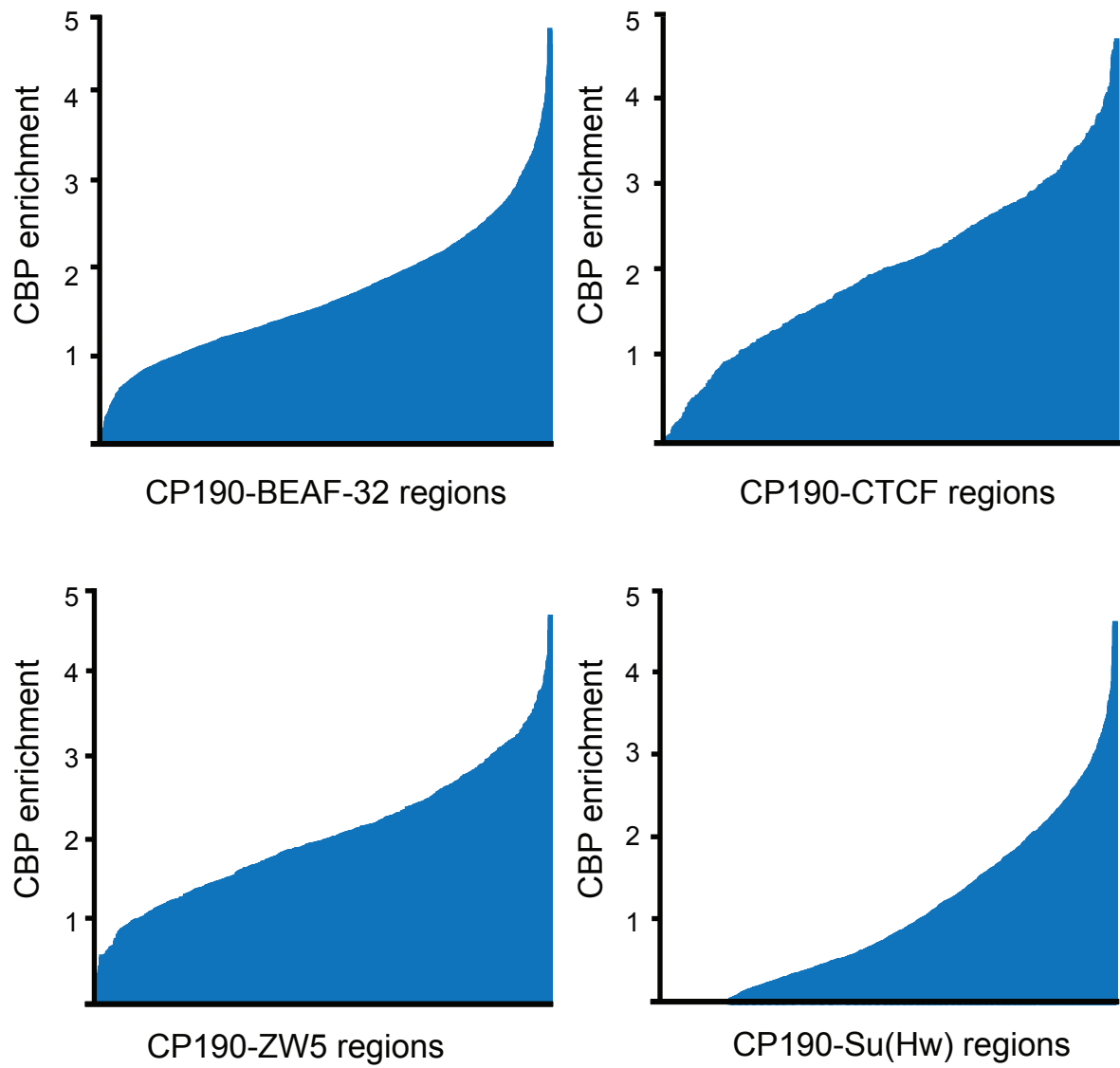

Supplement: Supplementary file 4 — 10.1186/s13072-015-0042-4 IP versus input enrichment of CBP in four classes of insulator regions. Regions are sorted by CBP enrichment. [file 13072_2015_42_MOESM4_ESM.pdf]
